# Eco-epidemiological analysis of rickettsial seropositivity in rural areas of Colombia: A multilevel approach

**DOI:** 10.1371/journal.pntd.0005892

**Published:** 2017-09-18

**Authors:** Juan C. Quintero V., Luis E. Paternina T., Alexander Uribe Y., Carlos Muskus, Marylin Hidalgo., Juliana Gil., Astrid V. Cienfuegos G., Lisardo Osorio Q., Carlos Rojas A.

**Affiliations:** 1 Grupo de Ciencias Veterinarias - Centauro, Facultad de Ciencias Agrarias, Universidad de Antioquia, Medellín, Colombia; 2 Grupo de Investigaciones Biomédicas, Universidad de Sucre, Sincelejo, Colombia; 3 Programa de Estudio y Control de Enfermedades Tropicales -PECET, Facultad de Medicina, Universidad de Antioquia, Medellín, Colombia; 4 Grupo de Investigación de Enfermedades Infecciosas, Departamento de Microbiología, Pontificia Universidad Javeriana, Bogotá D.C., Colombia; 5 Grupo de Investigación en Microbiología Básica y Aplicada, Escuela de Microbiología, Universidad de Antioquia, Medellín, Colombia; 6 Grupo de Investigación Salud y Ambiente, Facultad Nacional de Salud Pública, Universidad de Antioquia, Medellín, Colombia; 7 Grupo de Epidemiología, Facultad Nacional de Salud Pública, Universidad de Antioquia, Medellín, Colombia; University of California Davis, UNITED STATES

## Abstract

Rickettsiosis is a re-emergent infectious disease without epidemiological surveillance in Colombia. This disease is generally undiagnosed and several deadly outbreaks have been reported in the country in the last decade. The aim of this study is to analyze the eco-epidemiological aspects of rickettsial seropositivity in rural areas of Colombia where outbreaks of the disease were previously reported. A cross-sectional study, which included 597 people living in 246 households from nine hamlets in two municipalities of Colombia, was conducted from November 2015 to January 2016. The survey was conducted to collect sociodemographic and household characteristics (exposure) data. Blood samples were collected to determine the rickettsial seropositivity in humans, horses and dogs (IFA, cut-off = 1/128). In addition, infections by rickettsiae were detected in ticks from humans and animals by real-time PCR targeting *gltA* and *ompA* genes. Data was analyzed by weighted multilevel clog-log regression model using three levels (person, household and hamlets) and rickettsial seropositivity in humans was the main outcome. Overall prevalence of rickettsial seropositivity in humans was 25.62% (95%CI 22.11–29.12). Age in years (PR = 1.01 95%CI 1.01–1.02) and male sex (PR = 1.65 95%CI 1.43–1.90) were risk markers for rickettsial seropositivity. Working outdoors (PR = 1.20 95%CI 1.02–1.41), deforestation and forest fragmentation for agriculture use (PR = 1.75 95%CI 1.51–2.02), opossum in peridomiciliary area (PR = 1.56 95%CI 1.37–1.79) and a high proportion of seropositive domestic animals in the home (PR_20-40%_ vs _<20%_ = 2.28 95%CI 1.59–3.23 and PR_>40%_ vs _<20%_ = 3.14 95%CI 2.43–4.04) were associated with rickettsial seropositivity in humans. This study showed the presence of *Rickettsia* antibodies in human populations and domestic animals. In addition, different species of rickettsiae were detected in ticks collected from humans and animals. Our results highlighted the role of domestic animals as sentinels of rickettsial infection to identify areas at risk of transmission, and the importance of preventive measures aimed at curtailing deforestation and the fragmentation of forests as a way of reducing the risk of transmission of emergent and re-emergent pathogens.

## Author summary

Rocky Mountain spotted fever is one of the main diseases transmitted by tick bites in Colombia. Studies examining rickettsial seropositivity in humans, potential vectors and amplifying hosts in regions where previous outbreaks occurred are necessary to highlight this disease in the differential diagnosis of febrile syndromes and to implement epidemiological surveillance programs. This study reveals several factors associated with rickettsial seropositivity, including working outdoors, practices related to deforestation and forest fragmentation, and the potential contact between humans and wild animals, such as opossums, that could be involved in the transmission cycle. In addition, it reveals the importance of domestic animals as sentinels of infection as well as the tick species acting as potential vectors of rickettsiae in human and domestic animals.

## Introduction

Rocky Mountain spotted fever (RMSF) is a neglected disease without epidemiological surveillance in Colombia. This disease accounted for several deadly outbreaks in the northwest and the center of the country with a case fatality rate of 26 to 75% [1–3]. Fatal cases were related to delay in doxycycline treatment, the recommended therapy when RMSF is clinically suspected in endemic areas [4]. Most of the RMSF cases in Antioquia department (i.e., state) have been detected by research projects [5].

Previous studies in regions where outbreaks occurred have shown the presence of rickettsial antibodies in the human population as well as in domestic and wild animals [5–8]. However, factors associated with pathogen transmission are largely unknown because RMSF cases are usually undiagnosed or included as fevers of unknown origin in hospital surveillance records. Furthermore, the disease is similar to other febrile syndromes such as Dengue and Leptospirosis [9,10].

Although *Rickettsia rickettsii* and *Rickettsia parkeri* have been isolated from ticks of the *Amblyomma* genus collected from dogs and cattle in endemic areas of Colombia [5,11,12], there is limited information in this country about the species of ticks infesting humans and the rickettsial infection status of the identified tick species. Surveillance studies that focus on human infestation and tick infection are useful to reduce the number of cases and case fatality rate in endemic areas, as have been shown by surveillance programs implemented in United States [13]. In addition, the inclusion of domestic animals as sentinels of the disease is an essential component of surveillance studies. In Brazil, several reports have shown higher rickettsial infection rates by *Rickettsia* in domestic animals in endemic areas compared to non-endemic areas [14,15].

Regions affected by RMSF outbreaks in northwest Colombia had high levels of unmet basic needs and are affected by violence perpetrated by illegal armed groups [16]. These factors cause migration, changes in land use, deforestation and forest fragmentation near to urban areas, which increase interactions among humans, wild animals and disease vectors [17,18].

Studies of infectious diseases with a complex life cycle should include human populations, agents, amplifying hosts and vectors to better understand the ecological relationships governing pathogen transmission. Furthermore, individual and cluster variables are required for a comprehensive analysis of factors affecting the presence of these diseases. Consequently, the aim of this study is to analyze eco-epidemiological aspects of rickettsial seropositivity in two municipalities in northwest Colombia where previous RMSF outbreaks have been reported. The study tests the hypothesis of whether ecological factors (human, wild and domestic animals relationships) and social factors (occupation, sex, age, land use, household characteristics) influence the prevalence of rickettsial seropositivity in humans.

## Materials and methods

### Study design

A cross-sectional study from November 2015 to January 2016 was conducted in nine hamlets from two localities of northwestern Colombia, Alto de Mulatos in the municipality of Turbo (8°08'12.5"N 76°33'01.7"W), and Las Changas in the municipality of Necoclí (8°32'52.5"N 76°34'23.7"W). Inclusion criteria were: persons of all ages and residents from the hamlets selected in the study, who agreed to participate in the study, and who signed the informed consent. People suspected to be affiliated with illegal armed groups were excluded from the study. Participants were recruited by house-to-house visits in each hamlet.

### Sample size

Nine hamlets were selected for convenience: five in Alto de Mulatos and four in Las Changas. Hamlet selection was based on ease of access to the hamlets, shorter distance to the urban center, public safety, number of households and ecological conditions favorable for rickettsiae transmission, such as the presence of domestic animals, opossums, wild or synanthropic rodents, and previous reports of humans bitten by ticks. A finite probabilistic complex sample was designed, in which the sample units were households within the nine hamlets and analysis units were people inhabiting the households. To obtain the sampling frame a population census was conducted in the nine hamlets and information about the number of people, sex and presence of domestic animals in each house was collected. A total of 461 households inhabited by 1915 people were registered in the census. The sample size calculations indicated that 208 households inhabited by 865 persons would suffice to detect associations with 95% level of confidence, considering 5% error and 41% expected prevalence of infection in humans [7]. Sample size was calculated in Epidat 4.0 [19]. Households within each hamlet were selected using probability proportional to size sampling. In addition, non-probability sampling of domestic animals was done in the nine hamlets to study the role of canines and equines as sentinels of infection.

### Detection of rickettsial seropositivity in humans and domestic animals

Blood samples from humans and animals were taken for the serological diagnosis of rickettsial seropositivity by indirect immunofluorescence assay (IFA). Slides with *R*. *rickettsii* antigens were used to detect IgG against rickettsiae. The main outcome of the study was rickettsial seropositivity in humans defined as positive titers at 1/128 as previously recommended in endemic areas [20].

Rickettsial seropositivity in domestic animals was also defined as a positive titer at 1/128. In addition, antibody titers against several rickettsial antigens were investigated in animals to determine the potential species circulating in the study area. Slides containing specific antigens for *Rickettsia amblyommatis*, *R*. *rickettsii* and *R*. *parkeri* were used. The potential species of *Rickettsia* was determined when the IgG titer against that antigen was at least four serial dilutions higher than the IgG titer of other species evaluated. Positive controls were obtained from patients and domestic animals diagnosed by Laboratorio de Ciencias Veterinarias-Centauro, Universidad de Antioquia and Laboratorio de Microbiología, Pontificia Universidad Javeriana.

### Detection of rickettsiae in ticks collected from humans and domestic animals

Ticks were collected from December 2015 to May 2016. Study participants were asked to collect ticks attached to their skin or attached to clothes and place them in containers with 70% ethanol. Ticks were removed directly from dogs and equines during three minutes of collection per animal and were placed in containers with 70% ethanol. Ticks collected from humans and domestic animals were identified using the morphological key by Barros-Battesti *et al*. [21].

DNA extraction of ticks from all stages was done using Thermo GeneJet DNA purification kit. The DNA of each individual tick or 5-ticks pools (when ticks were not engorged) was extracted. Engorged ticks (>10 mg) were cut in sterile petri dishes using a sterile scalpel blade and pools were prepared according to the same stage, sex or individual host.

To confirm the absence of PCR inhibitors amplification of the 12S mtDNA gene in all collected ticks was performed [22]. In addition, species of ticks infected by rickettsiae were confirmed by sequence analysis of the 12S mtDNA.

To detect infection by *Rickettsia* of the Spotted Fever Group in ticks a real-time PCR amplifying a 146 bp of *gltA* gene was designed (*gltA*-Forward 5’- GCTCTTCTCATCCTATGGCT-3’, *gltA*-Reverse 5’- AGACATTGCAGCGATGGTAG-3’ and 5’-56FAM-TGCGGCTGTCGGTTCTCTTGCGGCA-3BHQ_1–3’.). Reactions were done using FastStart Universal Probe Master Mix Roche in LightCycler 96 Real Time-PCR System. Positive samples by Real Time-PCR were confirmed using primers to detect larger fragments of *gltA* (401pb) and *ompA* (631pb) [23,24]. Positive control used in PCR was DNA extracted from *Rickettsia rhipicephali*, donated by Dr. Marcelo Labruna, Laboratório de Doenças Parasitárias of Universidade de São Paulo, Brazil.

### Phylogenetic analysis

Forward and reverse sequences of 12S mtDNA from ticks and *gltA* and *ompA* from rickettsiae were analyzed in Geneious 8.1.5 [25]. Sequences were aligned to reference sequences from tick species of *Rickettsia* of the Spotted Fever Group, and the best model for nucleotide substitution was selected by Bayesian Information Criteria in jModelTest 2.12 [26]. Bayesian phylogenetic analysis was performed using Markov Chain Monte Carlo (MCMC) sampling implemented in MrBayes 3.2.6 [27].

### Individual variables

The exposures of interest were occupation, age (years), time of residence in the area (years), travel routes to the place of work, education level, previous exposure to ticks and previous episodes of fever. Occupation was measured as previous occupation (within the last five years) and recent occupation (at the time of the administration of the questionnaire). The variable was categorized as outdoors (farmers, ranchers and day laborers) and indoors (the remaining occupations).

### Household variables

Attitudes and practices related to rickettsioses were investigated with survey data. Heads of households (male or female) were interviewed with a structured questionnaire. The questions related to those practices common among family members. Household variables included: types of floors, walls and roofs, vegetation in the area surrounding the house, location of the house, proximity among households, previous tick infestations, presence of domestic animals (canines, felines, birds, pigs, donkeys, mules and horses), presence of rodents and opossums. As well, there were questions related to household members involved in deforestation and forest fragmentation for agricultural purposes in the area. In addition, attitudes and practices regarding use of white clothes when working outdoors, use of long-sleeved shirts, protection against rodents, dog bathing, and personal cleaning of tick infestation.

### Hamlets variables

Proportion of rickettsial seropositivity in domestic animals (canines and equines) was estimated in each hamlet by dividing the number of infected animals over the total number of tested domestic animals. Proportions were categorized as less than 20%, between 20 to 40% and more than 40%. Categorization was guided by reports from Brazil, where more than 40% of animals were seropositive in endemic areas for human rickettsioses and less than 20% were seropositive in non-endemic areas [14,28].

### Statistical analysis

Median and interquartile ranges were used for the description of quantitative variables. The linearity assumption was confirmed to include quantitative variables in bivariate and multivariate models. Absolute and relative frequencies were used for the description of qualitative variables (dichotomous and polytomous variables). To estimate risk factors for rickettsial seropositivity a weighted multilevel clog-log regression model was used. The model included three levels: individuals within households, households within hamlets (model random effect) and hamlets (model random effect). The association between the main outcome (rickettsial seropositivity in humans) and variables at each level was evaluated (household- and hamlet level). Variables included in the multivariate model were those with p<0.25 in bivariate analysis. The multivariate analysis was done using the stepwise method.

The multilevel model of risk factors for rickettsial seropositivity in humans was weighted by the inverse probability of animal selection because animal sampling was not proportional in the hamlets. Confounders were evaluated in multivariate models and the best model explaining the outcome was selected according to Bayesian and Akaike’s Information Criteria (BIC and AIC, respectively). All analyses were performed in SAS 9.04.01 [29].

Prevalence ratio was calculated using the following formula [30]:

$$\frac{P_{1}}{P_{0}}=\left(1-e^{-\text{e}(b_{0}+b_{1}+\sum_{j=2}^{k-1}b_{j}X_{j}}\right)/(1-e^{-\text{e}(b_{0}+\sum_{j=2}^{k-1}b_{j}X_{j}})$$

Where b_0_ is the model intercept, b is the regression coefficient (b_1_; b_2_;…b_j_), X are covariables (X_1_; X_2_;…; X_k-1_), and *e* is the base of Napierian logarithms. Prevalence Ratios (PR), 95% confidence intervals and p-values are reported in the models.

### Ethics statement

The Committee of Ethics in Research (meeting of May 22, 2014) and the Committee for Animal Experimentation (meeting of June 10, 2014) of the Universidad de Antioquia approved the participation of humans and animals in this study, respectively. All adult participants (≥18 years) signed the informed consent. Children (<18 years) were enrolled in the study after parents or guardians signed the informed consent on their behalf. The animal protocol used in this study adhered to the Colombian Law 84 of 1989 regulating the protection of animals against suffering and pain in the Colombian territory.

### Accesion numbers

The nucleotide sequences of 12S mtDNA, *gltA* and *ompA* genes have been deposited in the GenBank database under the accession numbers MF004422, MF004423, MF004424 MF034492, MF034493, MF034494, MF034495, MF034496 and MF034497.

## Results

### Characteristics of study population

A total of 597 people inhabiting 246 households participated in the study. House sampling coverage was 100% with 18.27% over-sampling. The sampling coverage of people was 69.01% (597/865). The overall prevalence of rickettsial seropositivity in humans was 25.62% (153/597) (95%CI 22.11–29.12). The prevalence was 30.41% (104/342) in Las Changas and 19.22% (49/255) in Alto de Mulatos. Prevalence in hamlets varied from 5.88% (1/17) to 37.29% (22/59).

Females accounted for 60.8% (363/597) of the participants. Median age was 29.7 years (IQR 15.3–46.1) and the median of the residence time in the hamlets was 11 years (IQR 6–19). In addition, 27.30% (163/567) of the population had worked in the last five years or had recently worked outdoors. Most of the participants were bitten by ticks in the previous days or years from the date of sampling (92.29%, 551/597). Other characteristics of the study population are presented in S1 Table.

### Household characteristics

46.34% of the households had a least one seropositive person. From 246 households, 44.72% (110/246) had thatched roofs (palm or cane), 69.92% (172/246) had complete or partial zinc roofs, 4.07% had complete or partial tiled roofs and 5.69% had complete or partial wood roofs (S1 Table).

Complete or partial dirt soil was present in 72.36% (178/246) of households and 4.47% (11/246) had complete or partial tile floors. In 88.21% (217/246) of households walls were made totally or partially of wood and 31.71% (78/246) of households had complete or partial brick walls. Other household characteristics are presented in S1 Table.

### Proportion of rickettsial seropositivity in domestic animals

The overall proportion of rickettsial seropositivity in canines and equines was 34.03% (81/238). Hamlets from Las Changas had a higher proportion of seropositivity (48.52%, 66/136) compared to hamlets from Alto de Mulatos (14.71%, 15/102). The proportion of seropositivity in equines was 30.88% (42/136) and 38.23% (39/102) in canines. Antibodies titers against *R*. *rickettsii* antigens in domestic animals from both localities were variable, ranging from 1/128 to 1/16384.

Analysis of titers against rickettsial antigens showed *R*. *amblyommatis* probably infected two horses, one donkey and one dog from Las Changas. *R*. *rickettsii* probably infected six donkeys and two horses from Las Changas and two mules from Alto de Mulatos. Finally, *R*. *parkeri* probably infected one dog from Alto de Mulatos (Table 1).

**Table 1:** Results of IgG antibodies titers against *R*. *rickettsii*, *R*. *parkeri* y *R*. *amblyommatis* antigens in equine and canine serum samples.

Serum sample	Las Changas (Animal species)	Alto de Mulatos (Animal species)	Antibody titers			Potential circulating species of *Rickettsia*
			*R*. *rickettsii*	*R*. *parkeri*	*R*. *amblyommatis*	
UC Las Changas 3*	*Equus caballus*		256	128	1024	*R*. *amblyommatis*
UC Las Changas 21*	*Eqqus asinus*		16384	<8192	<8192	*R*. *rickettsii*
La Unión 51	*Equus caballus*		2048	<1024	<1024	*R*. *rickettsii*
La Unión 78	*Eqqus asinus*		2048	<1024	<1024	*R*. *rickettsii*
La Salada 84	*Eqqus asinus*		4096	<2048	<2048	*R*. *rickettsii*
La Salada 87	*Eqqus asinus*		4096	<2048	<2048	*R*. *rickettsii*
La Salada 97	*Eqqus asinus*		1024	<512	<512	*R*. *rickettsii*
El Cativo 121	*Equus caballus*		2048	<1024	<1024	*R*. *rickettsii*
El Cativo 122	*Eqqus asinus*		16384	<8192	<8192	*R*. *rickettsii*
El Cativo 128	*Eqqus asinus*		128	<64	1024	*R*. *amblyommatis*
El Cativo 137	*Equus caballus*		1024	512	4096	*R*. *amblyommatis*
UC Alto de Mulatos 140*		*E*. *asinus x E*. *caballus*	1024	<512	<512	*R*. *rickettsii*
UC Alto de Mulatos 143*		*E*. *asinus x E*. *caballus*	1024	<512	<512	*R*. *rickettsii*
UC Las Changas 36	*Canis familiaris*		128	512	2048	*R*. *amblyommatis*
Quebrada del Medio 185		*Canis familiaris*	512	2048	512	*R*. *parkeri*

*UC: Urban Center

### Species of ticks collected from humans and tick infection by *Rickettsia*

A total of 458 ticks were collected, 49.78% (223/458) were *Amblyomma* nymphs, 19.65% (90/458) *Amblyomma* larvae, 16.16% (74/458) *Amblyomma cajennense* s.l. adults, 1.53% (7/458) *Amblyomma ovale* adults, 1.09% (5/458) *Amblyomma dissimile* adults, 8.30% (38/458) *Dermacentor nitens* nynphs, 2.40% (11/458) *Rhipicephalus microplus* and 0.66% (3/458) *Rhipicephalus sanguineus* adults (Table 2).

**Table 2:** Rickettsial infection in ticks collected from humans and domestic animals.

**Ticks species collected in humans (n = 458)**	**Number of ticks in Alto de Mulatos, n = 345 (%)**	**Number of ticks in Las Changas, n = 113 (%)**	**Infection by *Rickettsia* (*gltA* and *ompA*)**
*Amblyomma cajenense* s.l (Female)	8 (2,31)	22 (19.46)	0
*Amblyomma cajenense* s.l (Male)	13 (3,77)	31 (27,43)	0
*Amblyomma* sp (Nymph)	194 (56,23)	34 (30,08)	1/228^1^
*Amblyomma ovale* (Female)	0	3 (2,65)	0
*Amblyomma ovale* (Male)	0	4 (3,53)	0
*Amblyomma dissimile* (Female)	1 (0,29)	4 (3,53)	1/5^1^
*Amblyomma* sp (Larvae)	83 (24,05)	7 (6,19)	0
*Dermacentor nitens* (Male)	1 (0,29)	0	0
*Dermacentor nitens* (Nymph)	1 (0,29)	0	0
*Dermacentor nitens* (Larvae)	34 (9,85)	4 (3,53)	0
*Rhipicephalus (B) microplus* (Female)	0	2 (1,76)	0
*Rhipicephalus (B) microplus* (Male)	7 (2,03)	2 (1,76)	0
*Rhipicephalus sanguineus* (Female)	2 (0,58)	0	0
*Rhipicephalus sanguineus* (Male)	1 (0,29)	0	0
***Amblyomma* species collected in domestic animals (n = 433)**	**Number of ticks in Alto de Mulatos, n = 244 (%)**	**Number of ticks in Las Changas, n = 189 (%)**	**Infection by *Rickettsia* (*gltA* and *ompA*)**
*Amblyomma cajenense* s.l (Female)	38 (15,57)	53 (28,04)	0
*Amblyomma cajenense* s.l (Male)	27 (11,06)	15 (7,93)	0
*Amblyomma cajenense* s.l (Nymph)	111 (45,49)	26 (13,75)	0
*Amblyomma ovale* (Female)	16 (6,55)	13 (6,87)	3/29^2^^,^^3^
*Amblyomma ovale* (Male)	7 (2,86)	32 (16,93)	7/39^2^^,^^4^
*Amblyomma* (Larvae)	45 (18,44)	50 (26,45)	0

^1.^ The infected ticks only detected in Las Changas.

^2.^ Infected ticks were collected in canine from Las Changas.

^3.^ Pools of three female ticks.

^4.^ Two pools (one of two males and one of five male ticks).

**Fig 1:**
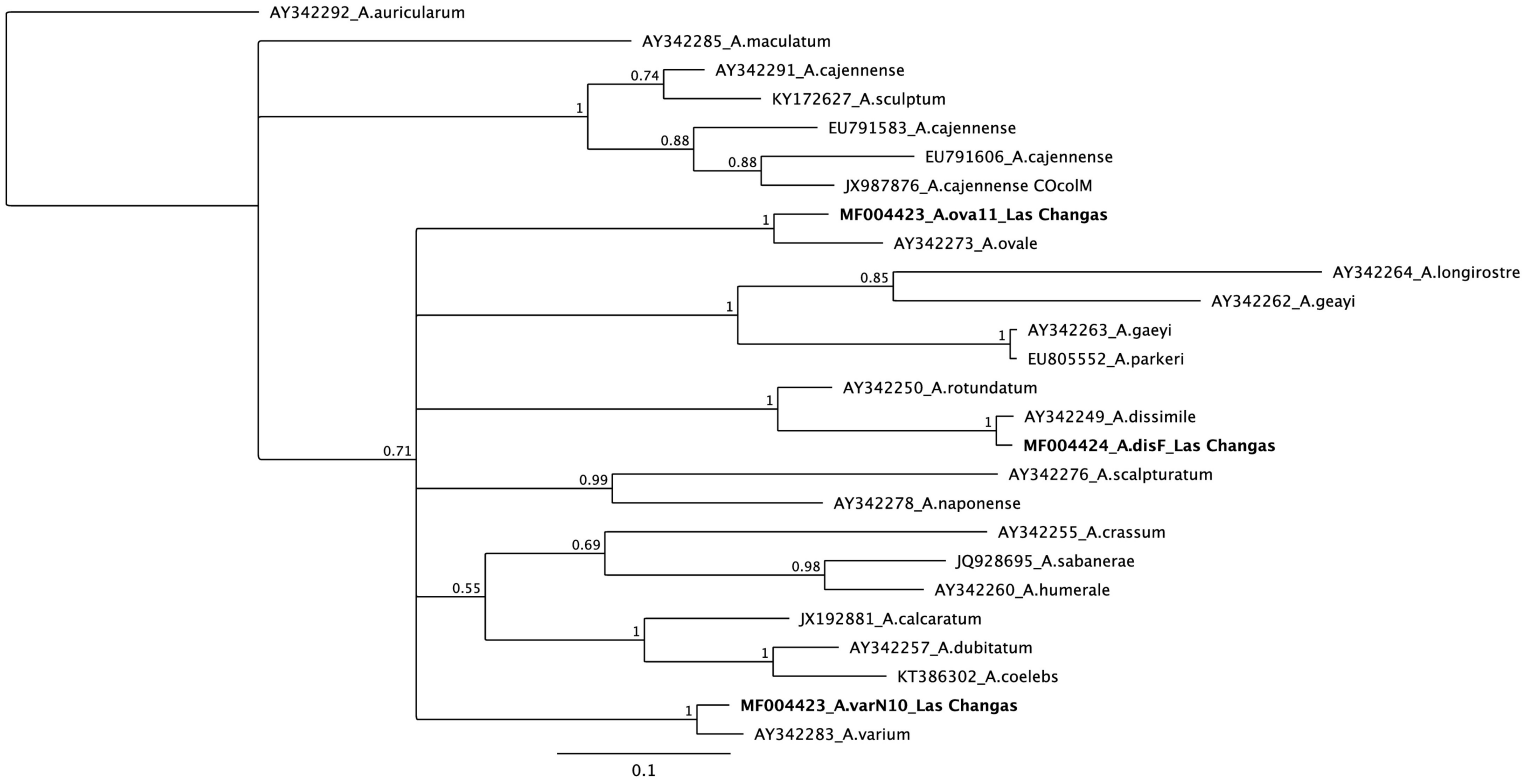
Bayesian phylogenetic analysis of 12S mtDNA (HKY+I+G model).

**Fig 2:**
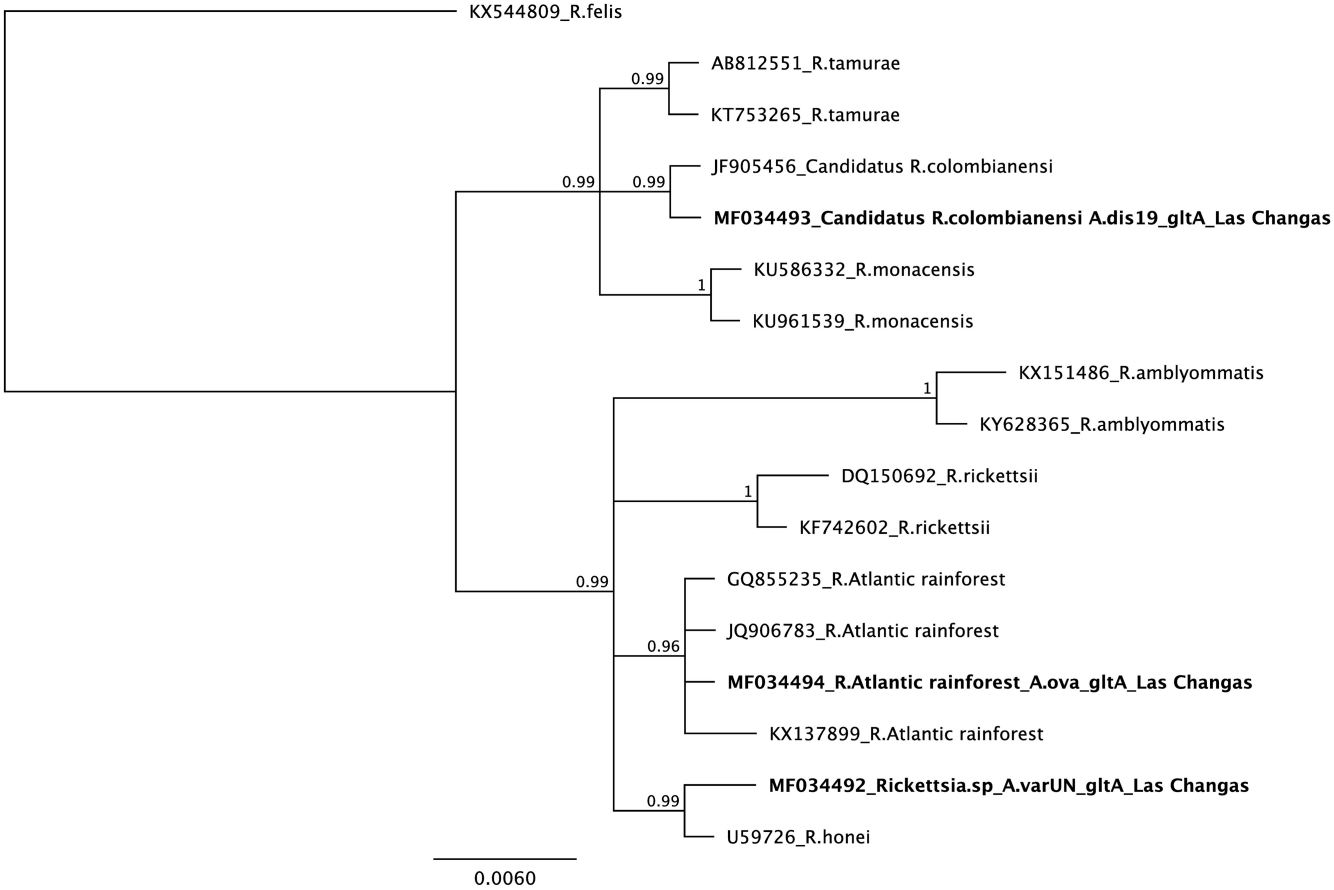
Bayesian phylogenetic analysis of *gltA* (TPM1uf model).

**Fig 3:**
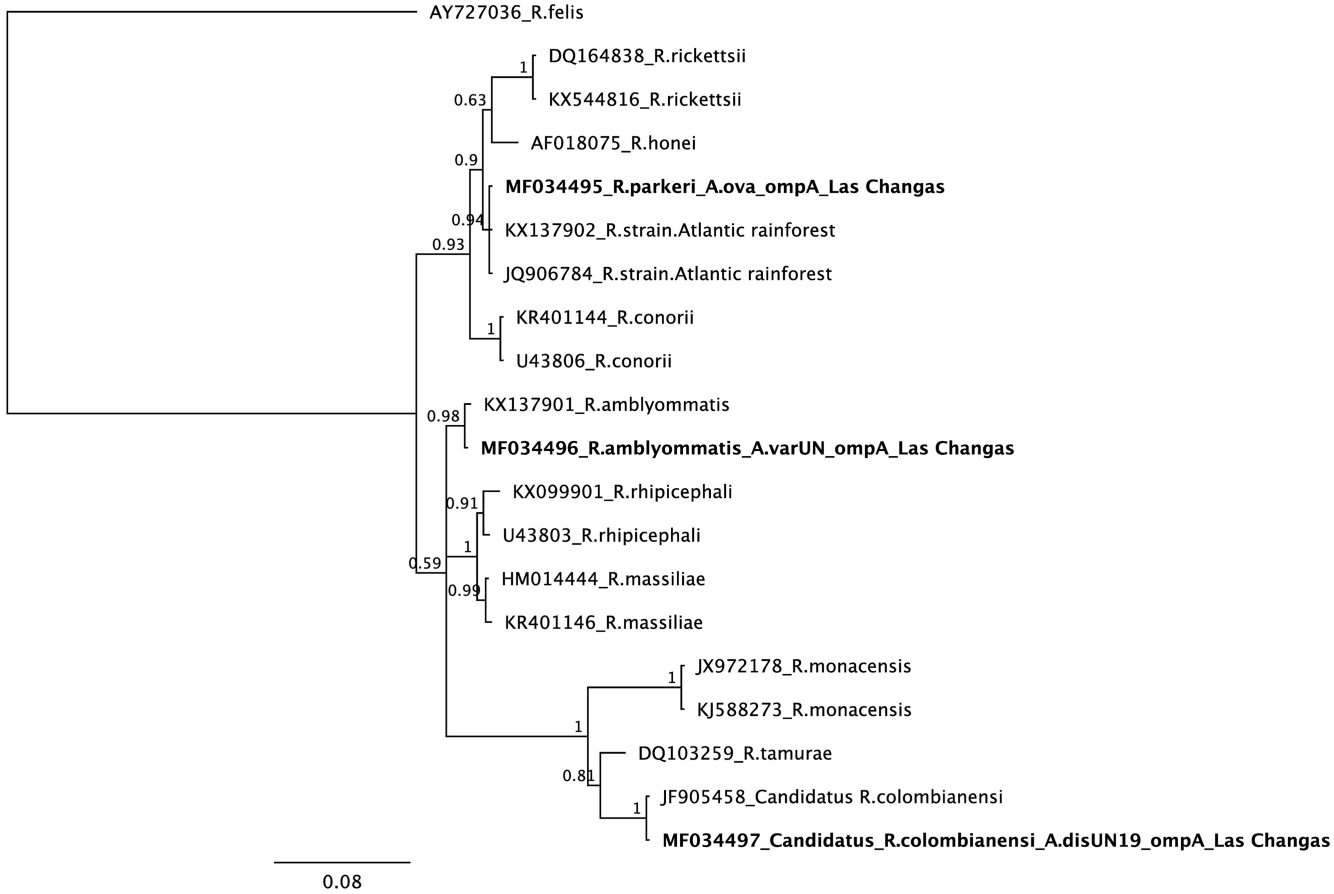
Bayesian phylogenetic analysis of *ompA* (TPM3uf+I model).

Two ticks collected from humans in Las Changas were infected by *Rickettsia*. One tick biting a person was closely related to *Amblyomma varium* in the Bayesian phylogenetic analysis of 12S mtDNA with 100% posterior probability support. Phylogenetic analysis of rickettsial *gltA* showed a close relationship to *Rickettsia honei* (99% posterior probability support), while *ompA* showed close a relationship to *R*. *amblyommatis* (98% posterior probability support). The second tick was attached to the clothes of one person and it showed a close relationship to *A*. *dissimile* by 12S mtDNA (100% posterior probability support) (Fig 1). Sequence analysis of *gltA* and *ompA* showed high similarity to Candidatus *Rickettsia colombianensi* (99 and 100% posterior probability support, respectively) (Figs 2 and 3, respectively).

### Species of ticks collected in domestic animals and tick infection by *Rickettsia*

A total of 433 ticks from *Amblyomma* genus were collected in Alto de Mulatos and Las Changas in January 2016. Identification of ticks revealed 133 (30.72%, 133/433) *A*. *cajennense* adults, 68 (15.70%, 68/433) *A*. *ovale* adults, 137 (31.64%, 137/433) nymphs and 95 (21.94%, 95/433) larvae (Table 2).

Rickettsial infection was detected in three pools (two, three and five individuals) of *A*. *ovale* (100% posterior probability support in Bayesian analysis of 12S mtDNA) infesting dogs from Las Changas (Fig 1). The Bayesian phylogenetic analysis of *gltA* and *ompA* showed a close relationship to *Rickettsia* sp. strain Atlantic Rainforest with more than 90% posterior probability support (Figs 2 and 3, respectively).

### Bivariate and multivariate analysis

The individual level variables included in multivariate analyses were occupation, sex, age and time of residence in the area. The household level variables were deforestation and forest fragmentation for agriculture use and presence of opossum in peridomiciliary areas. Hamlets level variable was proportion of seropositivity in domestic animals (Table 3).

**Table 3:** Description of variables included in the multivariate analysis and results of bivariate analysis.

**Individual level (n = 597)**	**Alto de Mulatos (n = 255)**	**Las Changas (n = 342)**	**Seropositivity in Alto de Mulatos (n = 49)**	**Seropositivity in Las Changas (n = 104)**	**PR (95%CI) (n = 597)**	**p-value**
Age (years) Median (IQR)	27.4 (14.5–44.5)	30.9 (15.8–47.4)	33.5 (17.8–52.2)	40.25 (19.85–56.4)	1.02 (1.01–1.02)	<0.001
Time of residence in the hamlets (years) Median (IQR)	10 (5–16)	12 (7–23)	12 (6–17)	14 (5–31.5)	1.01 (1.00–1.02)	0.0125
Sex (Men)	42.75%	36.55%	57%	46.15%	1.63 (1.23–1.98)	0.0012
Working outdoors	18.43%	33.92%	34.69%	42.31%	1.77 (1.33–2.14)	0.0003
**Household level (n = 242)**	**Households in Alto de Mulatos (n = 103)**	**Households in Las Changas n = 143)**	**Households with seropositive people in Alto de Mulatos (n = 39)**	**Households with seropositive people in Las Changas (n = 80)**	**PR (95%CI) (n = 242)**	**p-value**
Deforestation for agriculture use	71.84%	48.25%	76.92%	61.25%	1.70 (1.12–2.22)	0.0139
Presence of opossum in peridomiciliary	41.18%	50.71%	43.59%	59.49%	1.49 (1.07–1.89)	0.0185
**Hamlets level (n = 9)**	**Alto de Mulatos**	**Las Changas**	**Seropositive in Alto de Mulatos**	**Seropositive in Las Changas**	**PR (95%CI) (n = 9)**	**p-value**
Total number of domestic animals	102	136	14.71%(15/102)	48.52%(66/136)	1.02 (1.01–1.03)	0.0066
Number of equine	64	71	10.93%(7/64)	49.29%(35/71)	1.02 (1.00–1.04)	0.0446
Number of canine	38	65	21.05%(8/38)	47.69%(31/65)	1.03 (1.00–1.05)	0.0253
**Proportion of seropositivity in domestic animals (%)**						
<20					1.00	
20–40					2.44 (1.30–3.47)	0.0068
>40					2.91 (1.68–3.74)	0.0004
**Proportion of seropositivity in equines (%)**						
<20					1.00	
20–40					2.44 (1.30–3.47)	0.0068
>40					2.91 (1.68–3.74)	0.0004
**Proportion of seropositivity in canines (%)**						
<20					1.00	
20–40					2.08 (1.06–3.03)	0.0334
>40					3.13 (1.68–3.70)	0.0008

A comparison of BIC and AIC criteria showed the best model explaining the outcome included the variables sex, age, occupation, deforestation for agriculture use, presence of opossum in peridomiciliary area and proportion of seropositivity in domestic animals (Model 3, Table 4).

**Table 4:** Multivariate mixed models for rickettsial seropositivity.

Variables	Null model		Model 1		Model 2		Model 3	
	PR (95%CI)	p-value	PR(95%CI)	p-value	PR(95%CI)	p-value	PR(95%CI)	p-value
**Individual level (n = 597)**								
Working outdoors			1.22 (1.04–1.43)	0.013	1.17 (1.00–1.38)	0.052	1.20 (1.02–1.41)	0.024
Sex (Men)			1.67 (1.46–1.92)	<0.001	1.67 (1.45–1.92)	<0.001	1.65 (1.43–1.90)	<0.001
Age (years)			1.02 (1.01–1.02)	<0.001	1.01 (1.01–1.02)	<0.001	1.01 (1.01–1.02)	<0.001
**Household level (n = 242)**								
Deforestation for agriculture use					1.57 (1.33–1.86)	<0.001	1.75 (1.51–2.02)	<0.001
Presence of opossum in peridomiciliary					1.53 (1.34–1.75)	<0.001	1.56 (1.37–1.79)	<0.001
**Hamlets level (n = 9)**								
Proportion of seropositivity in domestic animals (%)								
<20							1.00	
20–40							2.28 (1.59–3.25)	<0.001
>40							3.14 (2.43–4.07)	<0.001
Number of domestic animals							1.01 (1.00–1.02)	<0.001
***Random effect (Hamlets level)***								
Variance (Standard error)	0.413 (0.091)	<0.001	0.446 (0.097)	<0.001	0.550 (0.115)	<0.001	0(0)	.
***Random effect (Household level)***								
Variance (Standard error)	0.318 (0.073)	<0.001	0.287 (0.073)	<0.001	0.242 (0.069)	0.002	0.227 (0.068)	0.005
AIC*	10804.98		10569.3		10388.56		10185.62	
BIC*	10805.58		10570.49		10390.13		10187.79	

* The model with the smaller value of the information criterion is considered to be better.

Household and hamlets random effects significantly explained the variability in the outcome in models 1 and 2, and only household random effect significantly explained the variability in outcome in model 3 (Table 4).

Male sex and age in years were risk markers for rickettsial seropositivity. The prevalence of seropositivity was 1.65-fold higher in men compared to women (PR = 1.65 95%CI 1.43–1.90). For each additional year of age, the prevalence of seropositivity increases by 1% (PR = 1.01 95%CI 1.01–1.02). Working outdoors had 1.20-fold increase in the prevalence of seropositivity compared to working indoors (Model 3, Table 4). Sex and age in years confounded the association between working outdoors and rickettsial seropositivity (PR_adjusted (sex and age)_ = 1.22 95%CI 1.04–1.43; PR_crude_ = 1.77 95%CI 1.33–2.14) (Model 1 in Tables 4 and 3, respectively).

In addition, the prevalence of seropositivity was 1.75-fold higher in people using deforested lands for agriculture compared to people who did not deforest (PR = 1.75 95%CI 1.51–2.02) and 1.56-fold higher in households where opossums were reported in the peridomiciliary area compared to households without opossum reports (PR = 1.56 95%CI 1.37–1.79) (Model 3, Table 4).

Interestingly, the proportion of seropositivity in domestic animals was an indicator of the serological status in humans. The prevalence of seropositivity in humans was 2.28-fold higher in hamlets where the proportion of seropositivity in domestic animals was 20% to 40% compared to hamlets where the proportion was less than 20%. Similarly, the prevalence of seropositivity in humans was 3.1-fold higher in hamlets where the proportion of seropositivity in domestic animals was more than 40% compared to hamlets with less than 20% (PR_20-40% vs <20%_ = 2.28 95%CI 1.59–3.25 y PR_>40% vs <20%_ = 3.14 95%CI 2.43–4.07) (Model 3, Table 4).

## Discussion

Our results showed a lower prevalence of rickettsial seropositivity in humans (25.62%) than in a previous study conducted in the same region (35–41%) [7]. This study had been conducted only in urban centers from both localities, while in our study hamlets were also included. High proportions of seropositivity in humans and domestic animals were found in La Union, La Salada and El Cativo hamlets in Las Changas. These results were similar to previous reports in endemic areas of Brazil [14].

Our study identified several factors associated with rickettsial seropositivity. Among individual-level variables, working outdoors was a risk for rickettsial infection due to people having greater exposure to ticks infesting woodland and grasses. Of note, participants from Las Changas worked outdoors more frequently (33.92%) than participants from Alto de Mulatos (18.43%), and consequently this population was more exposed to tick infestation. Similarly, in North Carolina in United States, it has been reported that people working outdoors had a higher risk of tick infestation and rickettsial infection [31].

However, tick infestation could be also determined by factors influencing household infestation, e.g., household materials, presence of domestic animals and rodents, among others [32]. Most of the study participants (92.29%) had been bitten by ticks during their lifetime, either recently or several years prior to the time of the inclusion in the study (S1 Table). These findings suggest that the risk of infestation exists in peri and intra-domiciliary areas.

We also found male sex and age were risk markers for rickettsial seropositivity. In these hamlets, men usually have outdoor occupations (e.g. as farmers, ranchers or day laborers), which increases the probability of being exposed to ticks as aforementioned. Accordingly, 75.50% of males worked outdoors and 74.42% of females worked indoors. In addition, older people were more frequently infected than younger participants. Older people had been exposed to rickettsiae for a longer period of time, which increased the opportunity of becoming infected. Consequently, seropositive people had a median age of 37.1 years (IQR 19.3–52.8), compared to median age of 27.5 years (IQR 14.4–44.4) for seronegative participants.

Likewise, participants that once worked outdoors had a median age of 44.3 years (IQR 29.7–57.1), while participants that work or worked indoors had a median age of 22.7 years (IQR 13–41.4). These results are consistent with reports from Cameroon, where age was also a risk marker for infection with *R*. *africae*, a bacterium causing a clinical syndrome similar to *R*. *parkeri*. In this previous study, the odds of infection increased by 80% in people aged 36–45 years compared to 16–25 years [33].

Among household-level variables, deforestation for agricultural use and the presence of opossums in peri-domiciliary areas were risk factors for rickettsial seropositivity. Frequent deforestation and fragmentation as observed in both areas facilitates human-vector interactions in the forest edge zones. This phenomenon was also observed in Lyme disease on Rhode Island, where edge zones presented a higher risk of tick infestation and consequently a higher risk of pathogen transmission [34].

Recently, the association between forest fragmentation and rickettsial infection in canines and RMSF in humans was investigated in São Paulo. The study reported that areas impacted by urban settlements or farming had a higher number of human RMSF cases [35]. Forest fragmentation significantly reduces both diversity and species composition of mammals subsequently reducing their interactions. In such cases, ticks parasitize available hosts (canines or humans), influencing the risk of human exposure to rickettsiae [36–39]. For instance, *Amblyomma aureolatum*, whose main host is *Cerdocyon thous*, a carnivorous mammal present in non-fragmented forests [40], can parasitize canines when forests are fragmented and competition among mammals decreases [41–43]. In such cases, *A*. *aureolatum*, one of the main vectors of *R*. *rickettsii* in Brazil, could infest dwellings and peridomiciliary areas [35,44,45], and become a risk for rickettsial infection for humans. Similarly, our results showed that canines and equines were infested with *A*. *cajennense* s.l., a tick species frequently found infesting humans with a potential role in the transmission of rickettsiae in the study area.

Exposure to opossums increased the prevalence of rickettsial seropositivity suggesting a possible role in pathogen transmission. However, as the presence of opossums in peri-domiciliary was reported by the participants, information bias could exist. The role of marsupials as amplifying hosts of rickettsiae in Colombia is obscure, yet in Brazil the opossum *Didelphis aurita* can have rickettsemia for long periods of time and therefore can become a source of infection for several tick species [46]. Recently, a high proportion of seropositives were detected in opossum *Didelphis marsupialis* in a previous study conducted in the Urabá region, supporting the role of these mammals in the life cycle of rickettsiae [47].

In addition, at the hamlets-level proportion of seropositivity in equines and canines were associated to the prevalence of rickettsial seropositivity in humans. This information suggests equines and canines could be sentinels for rickettsial infections, although this role is still debated in Brazil spotted fever endemic zones [8,14,48–50]. Consequently, studying prevalence of seropositivity in domestic animals could detect new areas of transmission where RMSF have not been diagnosed or are undiagnosed because of the lack of surveillance systems. In addition, high antibody titers in asymptomatic sentinels such as equines could indicate recent infections [51]. In our study, high antibody titers (1/16,384) detected in domestic animals suggesting that recent infections are occurring in the region.

Additionally, antibody titers also revealed the circulation of *R*. *rickettsii*, *R*. *amblyommatis* and *R*. *parkeri* in domestic animals. Furthermore, two of these species (*R*. *amblyommatis* and *R*. *parkeri*) were also detected in ticks infesting canines and humans, indicating the potential transmission of these rickettsiae among hosts. Similarly, previous studies in Colombia have detected ticks infected by *R*. *amblyommatis*, *R*. *parkeri* and *R*. *rickettsi* [11,12,52], and the latter species also have been reported circulating in canines and equines in Brazil [8]. Of note, only RMSF cases by *R*. *rickettsii* have been reported in the region and therefore the potential role of other rickettsiae species detected in ticks in our study should be investigated.

In our study, most of the ticks infesting humans were from *Amblyomma* genus. Accordingly, most of households had bushes (91.46%, 225/246), pasture (51.22%, 126/246) or trees (86.18, 212/246) in the peridomiciliary area, which are the sites preferred by *Amblyomma* ticks [32]. Importantly, humans were infested mainly by nymphs, which are known to be the most anthropophilic stage. In Brazil the majority of rickettsioses cases occur in winter and spring, seasons related to peaks of nymph abundance and infestation, mainly of *A*. *cajennense* s.l. [32].

Unexpectedly, *R*. *amblyommatis* was detected in one nymph of *A*. *varium*. This finding could explain why new clinical cases caused by *R*. *rickettsii* have not been diagnosed in the Urabá region. It has been suggested that in the United States the case fatality rate of RMSF decreased because the expansion of *Amblyomma americanum*, the vector of *R*. *amblyommatis*. The disease caused by *R*. *amblyommatis* is usually mild and the antigenic cross-reaction with other rickettsiae from the spotted fever can protect against virulent species, such as *R*. *rickettsii* [53].

In this study, Candidatus *R*. *colombianensi* was detected in ticks potentially infesting humans. This finding is of acaralogical and epidemiological interest since the status of this species as pathogen is unknown. In previous studies, Candidatus *R*. *colombianensi* was detected in ticks collected from iguanas (*Iguana iguana*) [54,55] and in one nymph of *Amblyomma* collected in spiny rat (*Proechimys semiespinosus*) [5].

In addition, *R*. *parkeri* strain Atlantic Rainforest was the only species detected in ticks *A*. *ovale* from canines. This species has been implicated in human clinical cases in rural regions of Brazil, characterized by eschar, fever, rashes on the legs and arms, and muscular and joint pain [56]. In our study, *A*. *ovale* infested humans in both localities, suggesting the potential role of *R*. *parkeri* as a cause of disease.

This study has several limitations. First, we had a lower coverage of sampling in humans. Because not all the people in the selected households agreed to participate, a high risk of selection bias is present in the study. Gaining people’s confidence to participate in the research project was difficult due to the increasing violence, presence of illegal armed groups and migration in the Urabá region. Second, information regarding variables at the household level should be measured using questionnaires in each age group to confirm the information retrieved at this level. However, in this study it was considered that behaviors and habits are similar among household members as well as within hamlets and regions. Likewise, risk perception and health care are also shared among people depending where they have lived or grown-up [57]. Fourth, although the collection of ticks in humans was voluntary, information retrieved about species infesting humans is essential to gain knowledge of species involved in rickettsiae transmission. Of note, our results showed a rich diversity of tick species infesting humans, including ticks commonly infesting canines, equines, bovines, turtles and iguanas.

The moderate to high prevalence of rickettsial seropositivity in humans and animals suggests circulation of rickettsiae in both populations. To obtain a better insight on circulation of rickettsiae among humans, it is necessary to diagnose clinical cases of the disease or at least to estimate the incidence of infection in areas where RMSF cases have been reported. Most of the studies on rickettsiae in Colombia are descriptive or cross-sectional, which makes it difficult to interpret the temporality of the effects estimated [58–60].

In conclusion, our study showed that working outdoors is a major factor associated to rickettsial seropositivity, and is influenced by age and sex. In addition, our results highlighted the role of domestic animals as sentinels of disease in areas with circulation of rickettsiae. Furthermore, preventive measures to avoid deforestation and forest fragmentation should be taken in the region to decrease the risk of pathogen transmission.

Finally, new studies that seek to assess the strength of the evidence related to factors associated to RMSF cases are necessary to ensure the appropriate diagnosis and timely treatment of this neglected disease and consequently to decrease the case fatality rate caused by this disease.

## Supporting information

S1 Checklist STROBE checklist. (DOC)

S1 Table Bivariate analysis of household and individual variables included in the survey. (DOCX)
