# Supplementary material for: Eco-epidemiological analysis of rickettsial seropositivity in rural areas of Colombia: A multilevel approach
Source: PLoS Negl Trop Dis. 2017 Sep 18;11(9):e0005892. doi: 10.1371/journal.pntd.0005892 (PMC5619838; doi:10.1371/journal.pntd.0005892)
Supplement: S1 Table — (DOCX) [file pntd.0005892.s002.docx]

Supplementary table 1. Bivariate analysis of household and individual variables included in the survey.

| **Variables** | **Total**  **n=597**  **n(%)** | **Infected**  **n=153**  **n(%)** | **Non-infected**  **n=444**  **n(%)** | **PR (95% IC)** | ***p-value*** |
| --- | --- | --- | --- | --- | --- |
|  |  |  |  |  |  |
| **Roof material** |  |  |  |  |  |
| Zinc | 407 (68.17) | 96 (62.75) | 311 (70.05) | 0.88 (0.58-1.31) | 0.527 |
| Thatched | 292 (48.91) | 86 (56.21) | 206 (46.40) | 1.25 (0.78-1.93) | 0.346 |
| Wood | 36 (6.03) | 12 (7.84) | 24 (5.41) | 1.54 (0.83-2.64) | 0.166 |
| Tile | 23 (3.85) | 4 (2.61) | 19 (4.28) | 0.84 (0.30-2.11) | 0.727 |
| Cement | 6 (1.01) | 2 (1.31) | 4 (0.90) | 1.6 (0.34-4.26) | 0.556 |
| **Floor material** |  |  |  |  |  |
| Dirt soil | 441 (73.87) | 112 (73.20) | 329 (74.10) | 0.84 (0.56-1.23) | 0.374 |
| Cement | 253 (42.38) | 59 (38.56) | 194 (43.69) | 0.93 (0.63-1.35) | 0.723 |
| Wood | 43 (7.20) | 9 (5.88) | 34 (7.66) | 0.75 (0.36-1.47) | 0.415 |
| Tile | 27 (4.52) | 5 (3.27) | 22 (4.95) | 0.74 (0.29-1.73) | 0.509 |
| **Wall material** |  |  |  |  |  |
| Wood | 540 (90.45) | 139 (90.85) | 401 (90.32) | 0.98 (0.55-1.67) | 0.948 |
| Blocks | 175 (29.31) | 35 (22.88) | 140 (31.53) | 0.73 (0.48-1.09) | 0.125 |
| **Location of the house** |  |  |  |  |  |
| Rural | 304 (50.92) | 79 (51.63) | 225 (50.68) | 1.00 |  |
| Urban | 293 (49.08) | 74 (48.37) | 219 (49.32) | 0.97 (0.79-1.19) | 0.789 |
| **Proximity among houses** |  |  |  |  |  |
| Very near | 138 (23.12) | 27 (17.65) | 111 (25.00) | 1.00 |  |
| Near | 197 (33.00) | 62 (40.52) | 135 (30.41) | 1.57 (1.00-2.38) | 0.051 |
| Disperse | 147 (24.62) | 28 (18.30) | 119 (26.80) | 0.92 (0.50-1.64) | 0.790 |
| Very disperse | 115 (19.26) | 36 (25.53) | 79 (17.79) | 1.37 (0.73-2.44) | 0.319 |
| **Characteristics of peridomicilliary area** |  |  |  |  |  |
| Vegetation |  |  |  |  |  |
| Bush | 552 (92.46) | 140 (91.50) | 412 (92.79) | 1.02 (0.54-1.82) | 0.949 |
| Trees | 532 (89.11) | 138 (90.20) | 394 (88.74) | 0.89 (0.64-1.88) | 0.680 |
| Grass | 314 (52.60) | 76 (49.67) | 238 (53.60) | 0.98 (0.66-1.44) | 0.932 |
| Cultures |  |  |  |  |  |
| Cassava | 36 (6.03) | 12 (7.84) | 24 (5.41) | 1.24 (0.67-2.16) | 0.477 |
| Corn | 18 (3.02) | 3 (1.96) | 15 (3.38) | 0.47 (0.13-1.52) | 0.220 |
| Cacao | 17 (2.85) | 4 (2.61) | 13 (2.93) | 0.79 (0.28-2.02) | 0.652 |
| Tomato | 16 (2.68) | 6 (3.92) | 10 (2.25) | 1.66 (0.67-3.35) | 0.256 |
| Ñame | 10 (1.68) | 4 (2.61) | 6 (1.35) | 1.27 (0.37-3.29) | 0.685 |
| Animals |  |  |  |  |  |
| Poultry | 421 (70.52) | 108 (70.59) | 313 (70.50) | 0.99 (0.68-1.41) | 0.941 |
| Pigs | 288 (48.24) | 82 (53.59) | 206 (46.40) | 1.16 (0.81-1.64) | 0.418 |
| Donkey | 189 (31.66) | 58 (37.91) | 131 (29.50) | 1.17 (0.72-1.82) | 0.519 |
| Turkey | 168 (28.14) | 53 (34.64) | 115 (25.90) | 1.19 (0.80-1.71) | 0.382 |
| Horse | 150 (25.13) | 39 (25.49) | 111 (25.00) | 0.99 (0.66-1.45) | 0.957 |
| Mule | 78 (13.07) | 23 (15.03) | 55 (12.39) | 1.35 (0.84-2.09) | 0.210 |
| **Presence of animals in households** |  |  |  |  |  |
| Canines | 343 (57.45) | 92 (60.13) | 251 (56.53) | 1.05 (0.74-1.47) | 0.766 |
| Felines | 364 (60.97) | 92 (60.13) | 272 (61.26) | 0.92 (0.64-1.29) | 0.635 |
| Rats | 481 (80.57) | 122 (79.74) | 359 (80.86) | 0.96 (0.64-1.41) | 0.833 |
| **Ticks infesting households** | 383 (65.25) | 95 (62.50) | 288 (66.21) | 1.43 (0.75-2.58) | 0.275 |
| **Practices** |  |  |  |  |  |
| Use of white clothes for working outdoors | 157 (26.30) | 48 (31.37) | 109 (24.55) | 1.20 (0.81-1.72) | 0.353 |
| Use of long sleeved shirts for working outdoors | 456 (76.38) | 124 (81.05) | 332 (74.77) | 1.15 (0.73-1.78) | 0.534 |
| Tick elimination after working outdoors | 324 (54.27) | 81 (52.94) | 243 (54.73) | 0.91 (0.63-1.28) | 0.579 |
| Tick infestation | 551 (92.29) | 144 (23.13) | 9 (19.57) | 0.36 (0.58-1.31) | 0.448 |
| Use of any rodent elimination measures | 353 (59.13) | 101 (66.01) | 252 (56.76) | 1.20 (0.74-1.88) | 0.448 |
| **Education attained** |  |  |  |  |  |
| No formal education | 58 (9.72) | 22 (14.38) | 36 (8.11) | 1.00 |  |
| Complete primary | 89 (14.91) | 20 (13.07) | 69 (15.54) | 0.63 (0.35-1.12) | 0.117 |
| Incomplete primary | 201 (33.67) | 57 (37.25) | 144 (32.43) | 0.86 (0.53-1.36) | 0.531 |
| Complete secondary | 75 (12.56) | 20 (13.07) | 55 (12.39) | 0.82 (0.45-1.43) | 0.501 |
| Incomplete secondary | 154 (25.80) | 30 (19.48) | 124 (27.93) | 0.55 (0.31-0.95) | 0.029 |
| Tertiary | 20 (3.35) | 4 (2.61) | 16 (3.60) | 0.59 (0.20-1.55) | 0.311 |
